# Similarities and differences between patients included and excluded from a randomized clinical trial of vitamin d supplementation for improving glucose tolerance in prediabetes: interpreting broader applicability

**DOI:** 10.1186/s13063-015-0812-0

**Published:** 2015-07-15

**Authors:** Yuval Eisenberg, Hiba Mohiuddin, Karthik Cherukupally, Hassan Zaidi, Subhash Kukreja, Elena Barengolts

**Affiliations:** Department of Medicine, Section of Endocrinology, Diabetes, and Metabolism, University of Illinois at Chicago, 1819 West Polk Street, Chicago, IL 60612 USA; Department of Medicine, Section of Endocrinology, Jesse Brown VA Medical Center, 820 South Damen Avenue, Chicago, IL 60612 USA

**Keywords:** Pragmatic-explanatory Continuum Indicator Summary (PRECIS), diabetes, randomized control trial (RCT), vitamin D, prediabetes, exclusion criteria, inclusion criteria

## Abstract

**Background:**

Randomized Clinical Trial (RCT) designs range from highly selective resulting in lack of external validity to more inclusive, requiring large sample sizes to observe significant results. Few publications, however, have compared excluded to enrolled participants. We aimed to assess our trial’s design based on the effectiveness versus efficacy continuum using the Pragmatic-Explanatory Continuum Indicator Summary (PRECIS) tool and to compare included and excluded patients.

**Methods:**

Fifteen members of endocrinology section completed PRECIS for DIVA (D-Vitamin Intervention in VA) trial; an RCT evaluating vitamin D supplementation in improving dysglycemia in patients with prediabetes. Retrospective chart review compared subjects excluded (OUT) to those included (IN) in RCT. Student’s t and Chi-square tests were used to compare continuous and categorical variables. Additionally, multiple logistic regression was completed.

**Results:**

PRECIS scores were nearly universally pragmatic. 178 patients enrolled in DIVA trial were compared with 178 randomly selected patients excluded from study involvement. There was no significant difference between IN and OUT for the majority of the continuous and all of the categorical variables. Multivariate logistic regression identified only the A1c, HDL and Charlson Index as significant predictors of a participant’s inclusion or exclusion. There was higher HDL (51.3(13.9) versus 44.6(10.1), *P* = 0.001) and Charlson Index (2.85(1.6) versus 2.2(1.17), *P* = 0.001) for OUT versus IN groups.

Subanalysis of excluded patients in A1c range 5.7 to 6.9, had lower BMI (30.7(3.4) versus 32(2.7), *P* = 0.002) but higher HDL (mg/l: 49.7(11.8) versus 44.6(10.1), *P* = 0.001) and Charlson index (2.85(1.6) versus 2.2(1.17), *P* = 0.001) than included participants. Additionally, there was a trend towards higher rates of cancer (22.9 % versus 12.9 %, *P* = 0.033) but less psychiatric problems (56.2 % versus 72.5 %, *P* = 0.026) and thiazide diuretic use (18.1 % versus 29.8 %, *P* = 0.034).

**Conclusion:**

DIVA trial design appears to favor broad clinical applicability. The majority of objectively compared variables did not different between patients included and excluded from this RCT. Advice based on the evidence from this RCT may be applicable to a larger group of patients than those fitting inclusion/exclusion criteria alone.

**Trial registration:**

ClinicalTrials.gov NCT01375660 (registered 15 June 2011).

**Electronic supplementary material:**

The online version of this article (doi:10.1186/s13063-015-0812-0) contains supplementary material, which is available to authorized users.

## Background

Evidence-based advice depends on randomized clinical trials (RCT). These trials are considered “gold standard” for the development of guidelines including those for prediabetes and diabetes prevention and management [1].

RCT vary in their applicability to clinical practice. Many studies are designed with rigid criteria to identify a very specific population for a proposed intervention, which can result in a clinically relevant outcome. These results, however, are not easily transferrable to a larger pool of patients who would not have met criteria for inclusion. This lack of external validity is cited as the most frequent criticism of RCTs [2]. Other studies, attempting to be more inclusive, may fail to identify differences when more lax selection criteria are utilized.

This difference has been characterized as explanatory (efficacy) versus pragmatic (effectiveness) design [3]. An efficacy trial answers the question: “Can the intervention work?” and therefore it is also referred to as an explanatory trial. An effectiveness trial answers the question: “Does the intervention work when used in normal practice?” and, therefore, is also called a pragmatic trial [3]. Type 2 diabetes mellitus (T2DM) trials commonly, but not always, prove “efficacy” of diabetes treatment but tend to exclude many patients for a variety of reasons. For example, the VADT (Veterans Affairs Diabetes Trial) trial screened 20,027 patients but randomized 1,791 (8.9 %) and excluded 91.1 % [4]. A review of 41 National Institute of Health (NIH) RCTs noted an average exclusion rate of 73 % [5]. Safety is often cited as the reason for exclusion. However, pragmatism is lost when nearly 90 % of potential patients are excluded from evaluation of efficacy and safety in randomized clinical trials.

There are practically no publications dedicated to the comparison of patients included to those excluded from an RCT, and participants and non-participants are rarely compared [6].

The objective of this study was to assess the D-Vitamin Intervention in Veteran Administration (DIVA) trial design based on effectiveness versus efficacy continuum and to compare patients excluded to those included in the trial.

## Methods

The Pragmatic-Explanatory Continuum Indicator Summary (PRECIS) tool was created by Thorpe et al. [3]. The tool is intended for use by trial designers who wish to subject their protocol to review in order to evaluate it for the intended purpose and applicability. Ten domains are included: eligibility criteria, flexibility of the experimental and comparison interventions, practitioner expertise of the experimental and comparison interventions, follow-up intensity, primary outcome, participant compliance, practitioner adherence and analysis of the primary outcome. Each domain is graded from most pragmatic to most explanatory, and graphically represented by the PRECIS wheel as each of 10 spokes: points near the center represent the more explanatory and points near the rim represent the more pragmatic characteristics of the domain.

### Assessment of DIVA trial design

Application of the PRECIS tool to the DIVA trial was performed by members of the University of Illinois at Chicago Endocrinology section. In order to ensure familiarity with the PRECIS tool, the members of the endocrinology service were provided the original paper from Thorpe et al. [3], an example of previous application of PRECIS tool to assess trial design [7], and a sample wheel. The group then attended a brief presentation describing the concept of pragmatic versus explanatory trials and the domains involved in the PRECIS assessment. Finally, group members were given a brief review of DIVA trial design, the mini-abstract describing trial specifics, and were allowed to ask questions about the design to aid in completion of their wheel.

In completion of the PRECIS wheel, members were asked to provide individual numerical assessment of each domain and to plot their score on the provided wheel. Numerical assessment of each domain included 10 points with scores near 0 favoring efficacy research (that is, ideal environment) while scores near 10 favoring effectiveness research (that is, practical setting). The data was collated, and the average score for each domain was plotted to obtain the overall assessment.

### Retrospective chart review

We performed retrospective chart review to compare subjects excluded (OUT) to those included (IN) in the (DIVA Study NCT01375660).

The DIVA study is a randomized, double-blind, placebo controlled trial aimed at assessing the efficacy of vitamin D for improving glycemic control. African American male veterans with prediabetes (hemoglobin A1c (A1C) 5.7 to 6.4 and no antidiabetes medications) and hypovitaminosis D (25-hydroxyvitamin D (25OHD) 5 to 29 ng/ml) were randomly assigned to receive 12 months of either ergocalciferol (D2, 50,000 IU weekly) or placebo. Participants who were diagnosed with diabetes during screening or intervention (6.5 to 6.9 %) were also allowed in the study if they did not need to take antidiabetic medications and A1c remained <7 %. The primary outcome is changes in indices of insulin sensitivity as measured by Oral Glucose Insulin Sensitivity (OGIS) and Matsuda index based on results of Oral Glucose Tolerance Test (OGTT). Efficacy assessment will be based on intention-to-treat and per-protocol analyses.

The DIVA trial subjects provided an informed consent prior to study participation. The study was approved by the Office for the Protection of Research Subjects (OPRS) Institutional review Board (IRB) at The University of Illinois at Chicago: IRB #2011-0934.

#### Included and excluded participant data collection

**Fig. 1:**
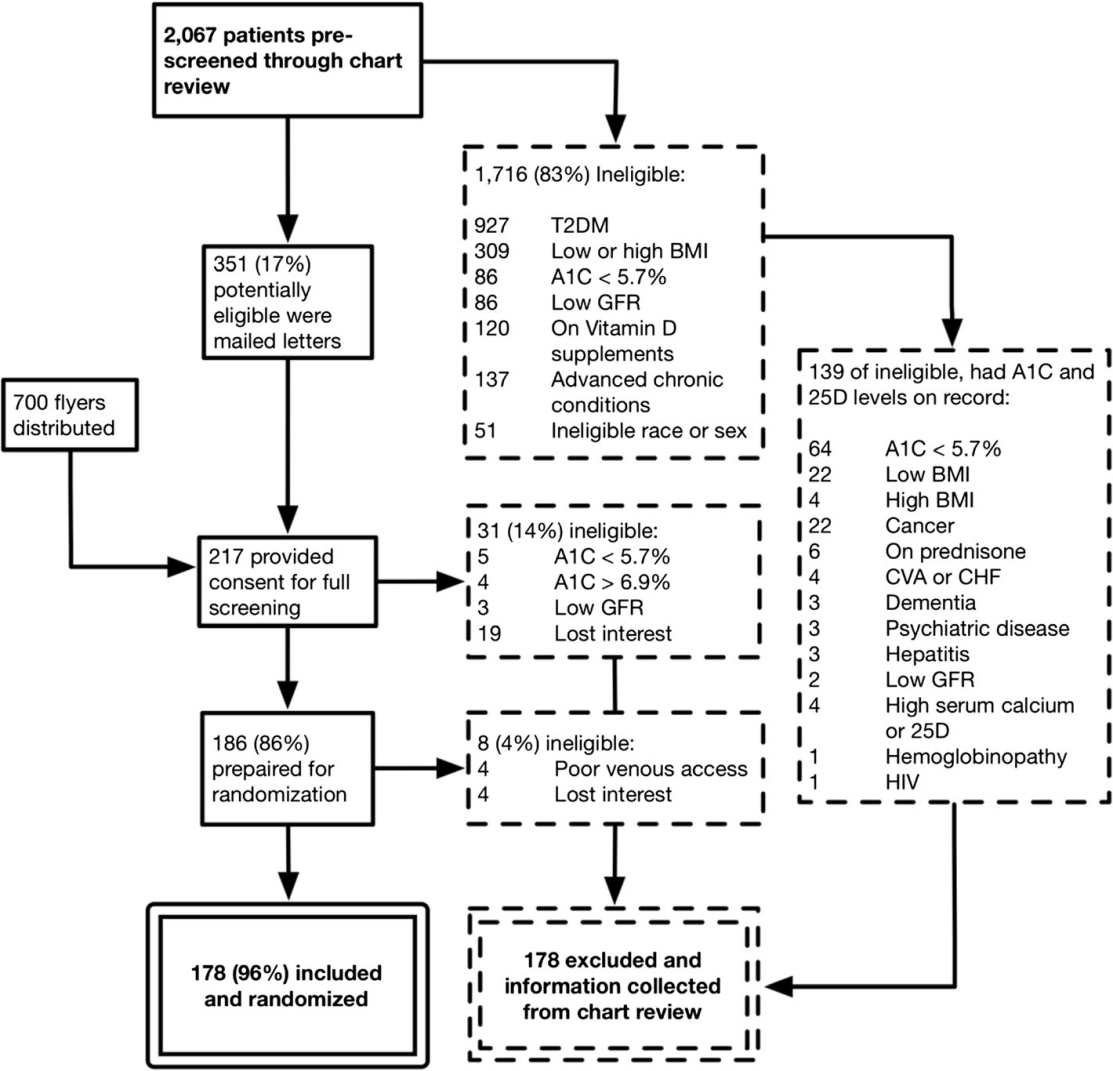
Exclusion flow sheet for D-Vitamin Intervention in Veterans’ Administration (DIVA) trial. Reason for ineligibility in dashed boxes. Of the 2,067 patients prescreened, 178 were enrolled and randomized (solid double box). An additional 178 of excluded patients were analyzed for comparison (dashed double box)

Recruitment for the DIVA trial started with 2,067 patients prescreened from Jesse Brown VA Medical Center (JBVAMC) electronic medical records for demographic criteria fitting the trial’s design: age, gender and race. Further screening resulted in majority ineligibility due to the presence of diabetes, weight/BMI, absence of prediabetes or advanced chronic medical conditions (Fig. 1). Those included (total 178 in June 2011) met the following criteria: age 35 to 85 years, BMI 28 to 39 kg/m2, A1C 5.7 to 6.9 % and circulating 25OHD level of 5 to 29 ng/ml. Major exclusion criteria were as follows: history of kidney stones, hyperparathyroidism, hypercalcemia, sarcoidosis, chronic kidney disease stage 3b or greater, or significant chronic medical condition that would interfere with study participation. A completed CONSORT checklist and flow diagram are available as supplementary files (see Additional file 1 and Additional file 2).

Of those ineligible for participation, a cohort of patients was chosen that would match the target population of the original DIVA trial described in above inclusion criteria. Of 217 subjects who came for full screening, 39 who were not randomized for the DIVA trial were included for this comparison study. Of 1,716 who were initially excluded, the inclusion and exclusion criteria for the current study were applied. The exclusions were as follows: type 2 diabetes, and ineligible sex or race. The inclusion criteria were as follows: African American men in whom a blood level for A1c and 25OHD was available within 12 months of the initial screening for DIVA trial. Based on exclusions, 1,098 were disqualified (Fig. 1). Of 618 remaining subjects, an additional 139 were randomly chosen until 178 total patients were found to match the number of those included in the DIVA trial.

Patient charts were reviewed retrospectively and data was collected from +/− 6mo from the time of initial exclusion (that is, time of prescreening, time of eligibility determination, withdrawal *etcetera*). Continuous variable data included: A1C, 25OHD level, estimated glomerular filtration rate (eGFR), lipid panel (cholesterol, LDL, HDL, and triglycerides), AST and ALT measures. Diagnoses recorded included: hypertension, hyperlipidemia, degenerative joint disease, cardiovascular disease, cancer and psychiatric problems. Additional information was collected on use of antihypertensive medications, thiazide diuretics, statins and vitamin D supplements. Finally, the age-adjusted Charlson index was calculated for all 356 patients based on the following calculations: 1 point for myocardial infarction, congestive heart failure, peripheral vascular disease, cerebrovascular disease, dementia, chronic obstruction pulmonary disease (COPD), connective tissue disease, peptic ulcer disease, uncomplicated diabetes and mild liver disease; 2 points for complicated diabetes, moderate to severe chronic kidney disease, hemiplegia, leukemia, lymphoma, solid tumor without metastasis;3 points for moderate to severe liver disease, and 6 points for metastatic solid tumors or AIDS [8]. Age adjustment added 1 point for every 10 years above age 40.

Blood samples measured specifically for study involvement or as reviewed in electronic medical records retrospectively were performed in the clinical laboratory applying laboratory standards of care and references. The analytical methods included ion-exchange high performance liquid chromatography (TOSOH g8 analyzer) for A1C and immunochemiluminometric assay (ICMA, DiaSorin LIAISON analyzer) for 25OHD.

### Statistics

#### PRECIS wheel assessment

Standardized descriptive statistics including measures of means and standard deviations were calculated for each point of PRECIS wheel. In addition, visual presentation of the PRECIS wheel from each participant was assessed qualitatively.

#### Included and excluded groups for DIVA trial assessment

Before statistical analysis, normal distribution and homogeneity of the variances were tested. Standardized descriptive statistics including measures of means and standard deviations for continuous variables were calculated. Categorical variables were summarized using the frequencies of the levels of the variables. Student’s t and Chi-square tests were used to compare continuous and categorical variables, respectively, between excluded and included participants. Statistical significance level was intended at *P* < 0.05, with Bonferroni correction for 22 variables setting *P* < 0.0023, using a two-sided test. A multiple stepwise logistic regression model was used to determine the predictors of the outcome included versus excluded groups, with all other characteristics used as the covariates. All variable with *P* < 0.2 from the ANOVA and Chi-squared test were input, and the stepwise backward model selection procedure was utilized. All statistical analyses were performed using SAS 9.2 statistical software (SAS Institute, NC)

## Results

**Fig. 2:**
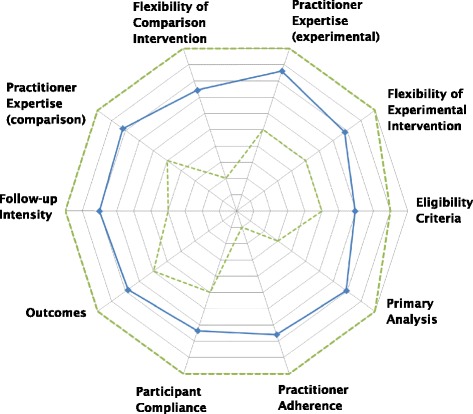
PRECIS wheel assessment of D-Vitamin Intervention in Veteran’s Administration (DIVA) trial. Mean score of 15 participants for each of 10 domains represented by a blue line. Maximum and minimum score given in each domain represented by outer and inner dotted line respectively

The 15-member group completing the PRECIS wheel assessment included three medical students, five endocrinology fellows, four medicine residents, and three endocrinology attending faculty. Members both involved (four members) or not directly involved (11 members) in the study completed the PRECIS assessment of the DIVA trial. The overall mean score for each domain is shown in the supplementary table (see Additional file 3). The scores were nearly universally on the pragmatic end of the spectrum, near the rim of the wheel (Fig. 2). The eligibility criteria domain was noted as the most explanatory (closest to the center), with an average score 6.9. Additionally, plotted were the most pragmatic and explanatory responses for each domain (dashed lines) (Fig. 2).

A total of 178 patients who were enrolled and randomized to participate in the DIVA trial between May 2011 and June 2012 were included for this analysis. An additional 178 patients who were excluded from involvement were randomly selected for in-depth profiling. There was no statistically significant difference between included and excluded patients for the majority of continuous and all of the reviewed categorical variables. There was a noted higher HDL (51.3 (13.9) versus 44.6 (10.1), *P* = 0.001) for excluded compared to included groups (Table 1). Additionally, the Charlson index was higher for excluded participants (2.85 (1.6) versus 2.2 (1.17), *P* = 0.001).

**Table 1:** Comparison of included and excluded participants

Characteristics	Included	Excluded	*P* value	Excluded A1c 5.7-6.9 %	*P* value
Number of Participants	178	178		105	
Continuous, Mean (SD)					
Age, yrs	59.1 (6.1)	60.1 (7.7)		62.1 (7.5)	
BMI, kg/m^2^	32.0 (2.7)	31.1 (3.5)		30.7 (3.4)	0.002
A1c, %	6.1 (0.2)	5.8 (0.5)		6.2 (0.3)	
25(OH)D, ng/ml	14.3 (5.1)	15.2 (5.5)		16.1 (5.7)	
eGFR, mL/min	85.7 (16.1)	87.1 (15.7)		83.4 (14.1)	
Cholesterol, mg/dl	182.2 (32.1)	173.3 (32.9)		175.8 (35.4)	
LDL, mg/dl	111.9 (27.0)	97.3 (31.2)		101.6 (34.0)	
HDL, mg/dl	44.6 (10.1)	51.3 (13.9)	0.001	49.7 (11.8)	0.001
TG, mg/dl	132.8 (59.4)	124.8 (58.7)		122.1 (58.4)	
AST, U/L	27.0 (10.3)	31.4 (15.6)		27.0 (11.1)	
ALT, U/L	45.8 (14.1)	50.8 (20.7)		47.5 (17.6)	
Charlson Index	2.20 (1.17)	2.85 (1.60)	0.001	3.11 (1.66)	0.001
Categorical, N (%)					
HTN	121 (68.0)	117 (65.7)		68 (64.8)	
Hyperlipidemia	97 (54.5)	84 (47.2)		55 (52.4)	
DJD	58 (32.6)	68 (38.2)		45 (42.9)	
CVD	29 (16.3)	31 (17.4)		23 (21.9)	
Cancer	23 (12.9)	34 (19.1)		24 (22.9)	
Psychiatric disorders	129 (72.5)	118 (66.3)		59 (56.2)	
Anti-HTN medications	108 (60.1)	103 (57.9)		61 (58.1)	
Thiazide diuretic use	53 (29.8)	37 (20.8)		19 (18.1)	
Statins	69 (38.8)	61 (34.3)		42 (40.0)	
Vit D supplements	30 (17.4)	48 (27.0)		25 (23.8)	

*P* values listed for significant findings compared to included group. Each excluded group was compared to included participants. *P* value deemed significant at *P* < 0.0023 after Bonferonni correction. BMI, body mass index; TG, triglycerides; HTN, hypertension; DJD, degenerative joint disease; CVD, cardiovascular disease

Excluded patients were then sub-classified for comparison with study participants in prediabetes or newly diagnosed diabetes range (A1c range 5.7 to 6.9 %). When selected for this A1c range, excluded patients had lower BMI (30.7 (3.4) versus 32 (2.7), *P* = 0.002) but higher HDL levels (mg/l: 49.7 (11.8) versus 44.6 (10.1), *P* = 0.001) and Charlson index (3.11 (1.66) versus 2.2 (1.17), *P* = 0.001), when compared with included participants. Additionally, although not reaching statistical significance, the excluded group was noted with a trend to higher incidence of cancer (22.9 % versus 12.9 %, *P* = 0.033) but lower incidence of psychiatric problems (56.2 % versus 72.5 %, *P* = 0.026) and thiazide diuretic use (18.1 % versus 29.8 %, *P* = 0.034) (Table 1).

The multivariate logistic regression showed that only the A1c, HDL and Charlson index were significant predictors of whether a participant would be included or excluded (Table 2).

**Table 2:** Determinants of inclusion or exclusion from the study^a^

Predictors^b^	Coefficient estimate	95 % confidence interval	*P* value
A1c	5.267	2.947, 9.413	<.0001
HDL	0.975	0.959, 0.991	0.0031
Charlson Index	0.701	0.581, 0.845	0.0002
Hyperlipidemia	1.045	0.630, 1.735	0.8639
Cancer	0.544	0.222, 1.333	0.1833
Psychiatric disorders	0.618	0.362, 1.055	0.0780
Thiazide diuretic use	0.634	0.362, 1.108	0.1097

Results reflect univariate analysis of 22 identified variables for *P* values <0.2 then subjected to multiple logistic regression

^a^Based on stepwise multiple logistic regression analysis for the whole group (n = 356)

^b^Predictors are values at baseline

## Discussion

At one extreme, a truly pragmatic trial tests treatment under real-life circumstances [7]. The results of such a trial would be presumed applicable to the broadest population possible and answer the question of intervention effectiveness [9]. A truly explanatory trial addresses an intervention performed under ideal conditions, attempting to control maximally for confounding variables. Clearly, no trial fits either of these models perfectly and, therefore, should be thought of as existing on a spectrum rather than as simply dichotomized [10].

This spectrum, visually represented by the PRECIS tool, was applied to an ongoing RCT. Our results show that both observers and study researchers believe the DIVA study to be a pragmatically designed trial. Although clearly subjective in nature, these results favor applicability to a broader population.

Several criticisms of this approach could be rendered. Knowledge of study design/methods is critical to one’s interpretation, but the very process of describing the trial inherently skews one’s opinion. Although well intentioned, bias certainly exists in self-assessment. Members of the review varied in their background and level of training (from medical student to attending physicians), which would also impact their ability to accurately assess a trial’s design. Additionally, the PRECIS tool has been helpful in modifying trial design [11], but has not been well studied as an assessment tool after trial initiation. Finally, alternative tools to distinguish efficacy from effectiveness studies could also have been utilized. Many tools have been proposed in the literature, albeit without clear validation, and have been utilized for review of already completed trials’ pragmatism [10].

RCTs always provide inclusion and exclusion criteria aimed at identifying a specific population of interest. This is requisite from a safety and ethical perspective, but inherently reduces broader applicability. Strictly speaking, applicability to an individual patient can be assessed by determining if that person would meet inclusion/exclusion criteria. Broader generalizability can also be assessed, from a population perspective, when large databases of patient characteristics exist; in centralized healthcare systems in many European countries. A recent epidemiologic study reviewed characteristics of 180,590 patients from Scotland with T2DM, and was able to evaluate inclusion/exclusion from seven major diabetes trials (ACCORD, ADVANCE, ProACTIVE, UKPDS 33/34, VADT, RECORD) [12]. When applied to Scottish diabetic patients, this analysis noted as few as 5 % of patients would have been included and that at most only 50 % would meet criteria. However, this approach may not clearly assess whether patients are statistically different from study participants by other clinically relevant variables.

Extensive baseline characteristics of treatment versus control groups are routinely provided to document similarity prior to intervention. Nearly every RCT presents clinical and outcome-relevant baseline data as reference within their results section. However, rarely in the literature are characteristics of participants who were excluded from a trial offered for comparison. In the ACTNOW trial, for example, nearly 1,900 patients underwent OGTT, extensive medical history, A1c, lipids, and urinary isoprostaglandins even before they were randomized to intervention versus placebo [13]. These data were not reported but may be useful for direct comparison with included subjects and to provide a reference for the population from which the researchers are drawing. The proposed benefit of providing or analyzing this data may be to identify objective limitations in applicability of a given trial’s results or conversely provide support for broader applicability despite the use of inclusion/exclusion criteria.

Here, we provide clinically relevant data to compare the large cohort of excluded patients to those enrolled in a randomized clinical trial. This objective data suggests that several clinical variables are statistically different between “In” and “Out” groups. This is not entirely surprising given that these populations were selected to be distinct (by inclusion/exclusion criteria). While statistical significance is important, the clinical significance of these differences should also be considered.

The excluded group had a higher burden of disease (by Charlson Index), lower BMI and overall higher HDL levels. Significant determinants of inclusion/exclusion into the trial were A1c, HDL level and Charlson index. These clinical differences are not entirely surprising in that they favor a “healthier” patient for trial enrollment. However, excluded participants also had fewer psychiatric problems (albeit not significant) and higher HDL levels, variables that would not be expected to be different. A potential explanation for the former may be a reflection of very high prevalence of psychiatric disorders in the veteran population and the frequency of care provided to them could have led to selection bias (for example, more likely to observe recruitment information). The explanation for the later is less clear but may be related to the insulin resistance and/or metabolically healthy obese phenotype in African Americans [14, 15].

Although there are some statistically different variables, the majority of the variables compared (18/22) were not significantly different between groups. This may suggest that results from the DIVA trial may in fact be applicable to a broader population. The PRECIS analysis additionally supports this assertion in describing the trial as more pragmatic.

## Conclusion

Our analysis shows that RCT, an accepted gold standard for evidence-based advice, has clinically relevant limitations. Although the notion exists that RCT involves highly selected patients, the comparison of included and excluded subjects is rarely done or reported in the literature. RCT vary in their pragmatism, which is important for physicians to understand regarding limitations of generalizing results to patients seen in clinical practice.

Analysis of the DIVA study design appears to favor pragmatic applicability as evidenced by the PRECIS wheel assessment. In addition, patients included and excluded from this RCT were similar by the majority of measured clinical characteristics. Advice based on the evidence from this RCT may be applicable to a broader population than simply those patients conforming to inclusion and exclusion criteria.

## Additional files

Additional file 1:
**Completed CONSORT checklist.**
Additional file 2:
**Completed CONSORT Flow Diagram.**
Additional file 3:
**PRECIS tool scores for each individual for all 10 domains.**

## Abbreviations

A1c: hemoglobin A1c
BMI: body mass index
DIVA: D-vitamin intervention in VA
OGTT: oral glucose tolerance test
PRECIS: pragmatic-explanatory continuum indicator summary
RCT: randomized controlled trial
T2DM: Type 2 diabetes mellitus
25OHD: 25 hydroxy vitamin D
