# Supplementary material for: Similarities and differences between patients included and excluded from a randomized clinical trial of vitamin d supplementation for improving glucose tolerance in prediabetes: interpreting broader applicability
Source: Trials. 2015 Jul 15;16:306. doi: 10.1186/s13063-015-0812-0 (PMC4502907; doi:10.1186/s13063-015-0812-0)
Supplement: Additional file 1: — Completed CONSORT checklist. [file 13063_2015_812_MOESM1_ESM.docx]

| **Member** | **Eligibility Criteria** | **Flex. Exp. Intervention** | **Practitioner Expertise (Experimental)** | **Flex. Comp. Intervention** | **Practitioner Expertise (Comparison)** | **Followup Intensity** | **Outcomes** | **Participant Compliance** | **Practitioner Adherence** | **Primary Analysis** |
| --- | --- | --- | --- | --- | --- | --- | --- | --- | --- | --- |

| #1 | 6 | 9 | 10 | 2 | 10 | 9 | 10 | 8 | 6 | 9 |
| --- | --- | --- | --- | --- | --- | --- | --- | --- | --- | --- |
| #2 | 5 | 6 | 9 | 9 | 9 | 5 | 6 | 8.5 | 8 | 6 |
| #3 | 8 | 7 | 9 | 7 | 9 | 10 | 6 | 7 | 10 | 8 |
| #4 | 5 | 7.5 | 5 | 7.5 | 5 | 8 | 7.5 | 8 | 8 | 8 |
| #5 | 6 | 8 | 7 | 8 | 7 | 9 | 8 | 9 | 8 | 7 |
| #6 | 8 | 9 | 8 | 9 | 6 | 7 | 9 | 10 | 7 | 10 |
| #7 | 9 | 10 | 10 | 10 | 5 | 7 | 10 | 10 | 10 | 10 |
| #8 | 8 | 10 | 10 | 8 | 8 | 10 | 8 | 10 | 8 | 10 |
| #9 | 8 | 8 | 10 | 8 | 10 | 7 | 7 | 5 | 10 | 6 |
| #10 | 5 | 6 | 8 | 5 | 8 | 9 | 7 | 5 | 5 | 8 |
| #11 | 8 | 8 | 5 | 9 | 8 | 9 | 10 | 6 | 5 | 8 |
| #12 | 9 | 9 | 10 | 9 | 10 | 9 | 7 | 6 | 1 | 3 |
| #13 | 6 | 7 | 9 | 7 | 9 | 9 | 7 | 6 | 9 | 9 |
| #14 | 6 | 8 | 9 | 8 | 9 | 4 | 7 | 6 | 9 | 7 |
| #15 | 7 | 5 | 10 | 5 | 10 | 8 | 8 | 6 | 10 | 10 |
| AVG | 6.9 | 7.8 | 8.6 | 7.4 | 8.2 | 8 | 7.8 | 7.4 | 7.6 | 7.9 |
| STDDEV | 1.4 | 1.5 | 1.7 | 2.1 | 1.7 | 1.7 | 1.3 | 1.8 | 2.5 | 1.9 |
