# Supplementary material for: Similarities and differences between patients included and excluded from a randomized clinical trial of vitamin d supplementation for improving glucose tolerance in prediabetes: interpreting broader applicability
Source: Trials. 2015 Jul 15;16:306. doi: 10.1186/s13063-015-0812-0 (PMC4502907; doi:10.1186/s13063-015-0812-0)
Supplement: Additional file 3: — PRECIS tool scores for each individual for all 10 domains. [file 13063_2015_812_MOESM3_ESM.docx]

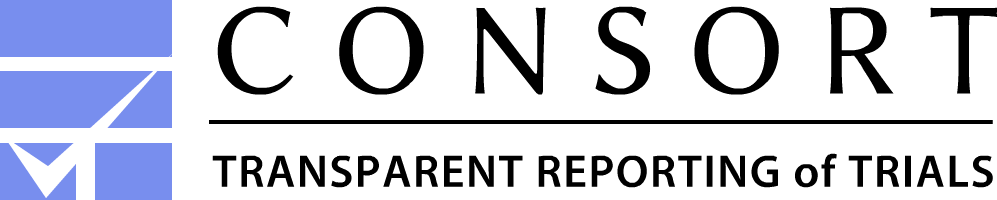


**CONSORT 2010 Flow Diagram**

## Follow-Up

Analysed (n=87 )
♦ Excluded from analysis (give reasons) (n=0)

## Analysis

Analysed (n=86 )
♦ Excluded from analysis (give reasons) (n=0)

Lost to follow-up (give reasons) (n=15, lost interest)

Discontinued intervention (give reasons) (n=1, hypercalcemia)

Lost to follow-up (give reasons) (n= 16, lost interest )

Discontinued intervention (give reasons) (n=0)

## Enrollment

Allocated to intervention (n=103)

♦ Received allocated intervention (n=103)

♦ Did not receive allocated intervention (give reasons) (n=0 )

## Allocation

Allocated to intervention (n=102)

♦ Received allocated intervention (n=102)

♦ Did not receive allocated intervention (give reasons) (n=0 )

Randomized (n=205)

Excluded (n=1862 )

♦  Not meeting inclusion criteria (n=1716)

♦  Declined to participate (n= 146 )

Assessed for eligibility (n=2067) )
